# IoT-Based Wireless System for Gait Kinetics Monitoring in Multi-Device Therapeutic Interventions

**DOI:** 10.3390/s24175799

**Published:** 2024-09-06

**Authors:** Christian Lang Rathke, Victor Costa de Andrade Pimentel, Pablo Javier Alsina, Caroline Cunha do Espírito Santo, André Felipe Oliveira de Azevedo Dantas

**Affiliations:** 1 Graduate Program in Neuroengineering, Edmond and Lily Safra International Institute of Neuroscience, Macaíba 59280-000, RN, Brazil; caroline.santo@isd.org.br (C.C.d.E.S.); andre.dantas@isd.org.br (A.F.O.d.A.D.); 2 Graduate Program in Electrical and Computer Engineering, Federal University of Rio Grande do Norte, Natal 59078-970, RN, Brazil; victor.andrade@ifrn.edu.br (V.C.d.A.P.); pablo@dca.ufrn.br (P.J.A.); 3 Department of Mechatronics, Federal Institute of Science, Education, and Technology of Rio Grande do Norte, Parnamirim Campus, Parnamirim 59143-455, RN, Brazil

**Keywords:** smart insoles, Internet of Things, gait analysis, biomechanical sensors, MQTT protocol

## Abstract

This study presents an IoT-based gait analysis system employing insole pressure sensors to assess gait kinetics. The system integrates piezoresistive sensors within a left foot insole, with data acquisition managed using an ESP32 board that communicates via Wi-Fi through an MQTT IoT framework. In this initial protocol study, we conducted a comparative analysis using the Zeno system, supported by PKMAS as the gold standard, to explore the correlation and agreement of data obtained from the insole system. Four volunteers (two males and two females, aged 24–28, without gait disorders) participated by walking along a 10 m Zeno system path, equipped with pressure sensors, while wearing the insole system. Vertical ground reaction force (vGRF) data were collected over four gait cycles. The preliminary results indicated a strong positive correlation (r = 0.87) between the insole and the reference system measurements. A Bland–Altman analysis further demonstrated a mean difference of approximately (0.011) between the two systems, suggesting a minimal yet significant bias. These findings suggest that piezoresistive sensors may offer a promising and cost-effective solution for gait disorder assessment and monitoring. However, operational factors such as high temperatures and sensor placement within the footwear can introduce noise or unwanted signal activation. The communication framework proved functional and reliable during this protocol, with plans for future expansion to multi-device applications. It is important to note that additional validation studies with larger sample sizes are required to confirm the system’s reliability and robustness for clinical and research applications.

## 1. Introduction

Human gait analysis is a complex science. Humans are the only mammals with natural bipedal walking, and it is still unknown why bipedal walking was selected over thousands of years of evolution [1]. Since the days of Aristotle (384–322 BCE), the fascination with unraveling the intricate mechanisms of locomotion has inspired countless researchers in an unrelenting quest for knowledge [2,3,4]. Gait can be affected by a variety of factors, including traumatic injuries or neurological disorders such as Parkinson’s disease and cerebral palsy, which profoundly affect the individuals’ quality of life [5,6]. Due to the need for tools that help professionals and individuals who suffer from some spectrum of movement disorders, the industry and researchers have dedicated their time to develop robust systems to evaluate, monitor, and assist in the treatment of movement disorders; among these systems, we can mention Fscan [7], Dynafoot2 [8], Flexinfit [9], Zeno(PKMAS) [10,11], and GAIT Rite [12].

A major barrier to disseminating these systems involves high acquisition costs and limitations in software customization for integration with multiple devices. Increasing technological advances have favored the development of compact devices capable of monitoring health in different contexts, and providing information during clinical assessments.

Among them, we can mention pressure sensors and inertial measurement units (IMU), used to evaluate gait by capturing data on plantar pressure distribution, center of pressure (COP), vertical ground reaction forces (vGRF), and spatiotemporal variables involved in gait cycle characterization [13,14,15,16,17]. Vertical ground reaction forces (vGRFs) are the forces between the foot and ground that can be obtained by wearable sensors and are considered the main measurement in kinetic analysis [18]. Measuring vertical ground reaction forces (vGRFs) during walking is useful for rehabilitation, early diagnosis, fall prevention, and in characterizing the gait cycles, as applied in different studies [19,20,21,22,23].

This study aims to develop and test a low-cost insole system, based on flexible sensors, that captures vertical ground reaction force (vGRF) data during walking in a multi-device integrated services approach through a healthcare IoT platform infrastructure. The system identifies the support phase of the gait cycle and its sub-phases, including the initial contact, loading response, middle support, and terminal support. In addition to the developed system, we added a proposal for a communication framework that we developed and applied in protocols based on multiple devices.

The MQTT protocol has been widely used in various research scenarios involving wearable devices due to its practicality and out-of-the-lab application [24,25,26]. An MQTT-based framework is available in our open source repository on GitHub named “Neurodevices” (accessed on 20 August 2024) which offers services for creating an integrated network with other devices during data collection. Devices such as electrical stimulators and inertial measurement devices were previously developed in our Neuroengineering Laboratory [27,28] at Santos Dumont Institute.

The results with devices were compared, and a Pearson correlation coefficient was computed for the data collected from both systems, for all individuals after a 10-m-long walk. Bland–Altman plots were obtained to evaluate the agreement between measurements. The initial and final two meters were considered acceleration and deceleration stretches and excluded from the sample. The main contributions of this work involve the use of low-cost materials in the search for an accessible and functional alternative to the existing commercial systems, exploring new network protocols between devices, favoring multi-modal assessments and therapies in the rehabilitation process, and providing a perspective on the inclusion of its use in the Brazilian Health Unified System (SUS).

## 2. Materials and Methods

### 2.1. Statement of Human and Animal Rights

The study is part of a research project submitted to the Ethics Committee of the Santos Dumont Institute under the reference number C.A.A.E. 53127921.2.0000.0129.

### 2.2. Subject and Study Design

This work had a cross-sectional observational design and involved four healthy individuals between 24 and 28 years old. A previous history of injuries or dysfunctions in the lower limbs was used as an exclusion criterion. The study was conducted at the International Institute of Neuroscience, Edmond and Lilly Safra, of the Santos Dumont Institute, located in Macaíba, Rio Grande do Norte, Brazil.

### 2.3. Insole Pressure Sensor

**Figure 1:**
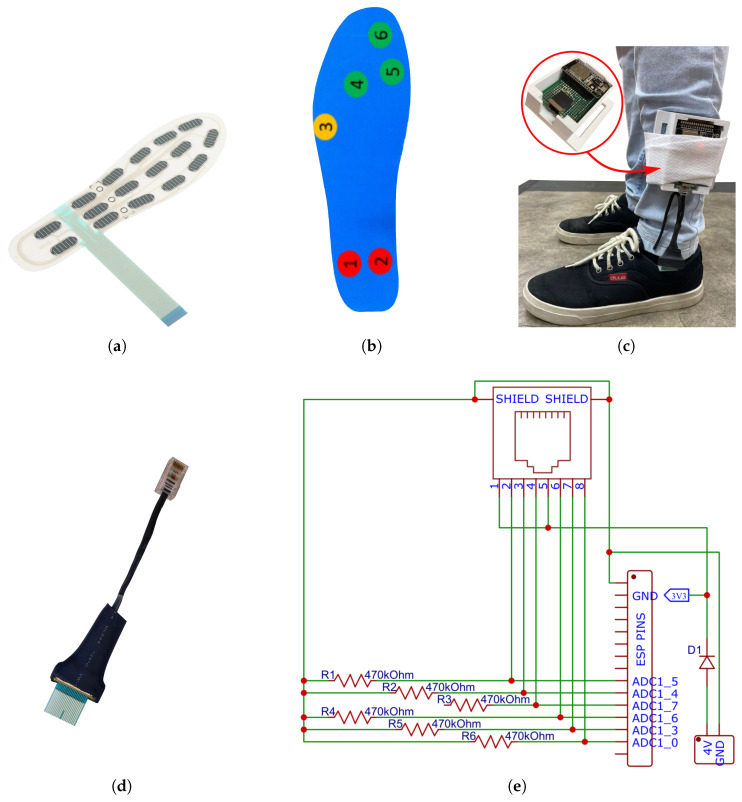
General components of the system. (**a**) Insole film sensor, (**b**) EVA lining, (**c**) prototype in use, (**d**) 8-wire Ethernet RJ-45 to FPC adapter, (**e**) electric diagram. Source: Authors.

In this subsection, we explore the materials applied to the study. Initially, the commercial insole pressure sensor FS-INS-16Z [29] (Shenzhen LEGACT Technology Co., Ltd, Shenzhen, China) was used, as shown in Figure 1a. We selected materials available in our laboratory by convenience, including low-cost perforated circuit boards and 3D printed polymers (ABS), and we reused materials from similar projects.

The FS-INS-16Z is made of polyester film and has 16 independent sensing regions (pressure sensors) with one output terminal each. It also includes two common and two null terminals, for a total of 20 terminals. The insole foot size is 41 according to the Chinese standard (CHN), which is equivalent to size 8 for the American (USA) and 38 for the Brazilian (BRA) standards. The insole operational specification is depicted in Table 1.

An ESP32-DevKitC (Espressif Systems Co., Ltd, Shanghai, China) was used to acquire the data from six sensors at a 120 Hz sample rate each and transmit them wirelessly via Wi-Fi. In addition, we used a nominal 3.7 V (800 mAh) battery that can be recharged via USB together with the system.

For greater comfort and protection, we used a 5 mm thick EVA lining, as shown in Figure 1b, attached to the polyester film insole without compromising the characteristics of the sensor. Six specific sensors were selected from the insole since we had a limitation of only six analog input pins available on the ESP32 board. Figure 1c shows the insole attached to the acquisition board and worn by a subject standing over the Zeno^®^ pathway (Protokinetics, LLC, Exton, PA, USA).

The ProtoKinetics Zeno^®^ Walkway Gait Analysis System is based on a sensor array for capturing plantar pressure, and based on this, a series of features were extracted from the data. According to the manufacturer, the device has different length and width configurations, which offers benefits concerning adaptation in diverse clinical environments. In addition to the gait analysis functionalities, the Zeno^®^ can also assess balance and a variety of movement protocols. However, this platform has limitations regarding its dimensions, hard installation procedures, and high cost; hence, it must be used only in a controlled environment, i.e., inside the lab.

### 2.4. Microcontroller and Circuit Prototyping

The circuit built for data acquisition consists of resistors (470 KΩ) connected in series with each insole sensor terminal to form voltage dividers connected to the ESP32 input pins. The voltage variation over the resistors is captured by the analog input ports of the microcontroller, as shown in the schematic illustrated in Figure 1e.

To perform validation measurements, the ESP32-DevkitC (accessed on 20 July 2024) development board was used, whose specification is configured to work with some of its analog ports specifically restricted for Wi-Fi communication purposes. Consequently, the experiment was constrained to reading data from six insole pressure sensors. These sensors were strategically placed, as designated by a physiotherapist, in the extremities of the feet, covering the hallux, metatarsus (first, second, and fifth), and calcaneus, as illustrated in Figure 1b.

Furthermore, an 8-wire Ethernet RJ-45 to FPC adapter shown in Figure 1d was constructed to provide an interface that provides power source from the acquisition board to the insole sensors and delivers insole output voltage signals to the acquisition board, physically connecting the flat pins of the insole to the ESP available analog inputs.

### 2.5. Communication Protocol

An ESP32 microcontroller Wi-Fi module was used to establish an MQTT connection with a client. We utilized a Python script to save the raw data into a CSV file. The MQTT protocol is a lightweight energy-efficient communication protocol commonly used to exchange messages between IoT devices.

**Figure 2:**
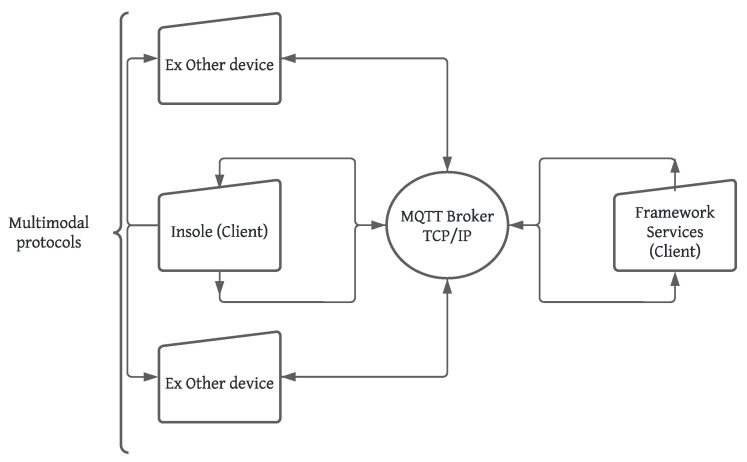
Proposed communication structure. Source: authors.

**Figure 3:**
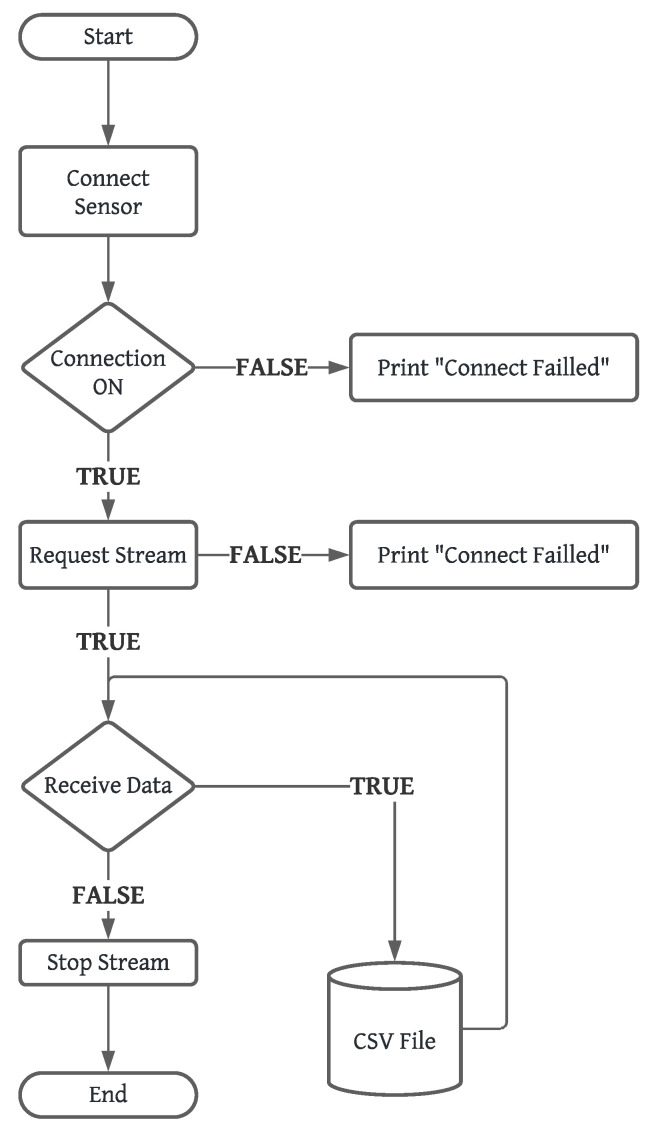
Diagram for MQTT data request. Source: authors.

The MQTT broker enabled bidirectional communication between clients, as illustrated in Figure 2. In this case, the insole acquisition data system acts as an MQTT client responsible for sending (publishing) messages containing data from the insole’s pressure sensor. These messages are published on MQTT topics, and MQTT subscribers receive data messages from the insole. The diagram in Figure 3 illustrates the operation of the communication system and the data capture performed by the Python MQTT clients using the paho-mqtt library.

**Figure 4:**
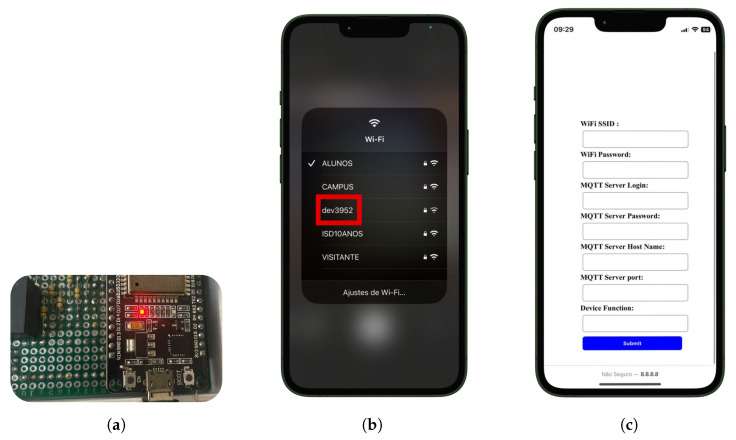
Representation of device configuration screens. (**a**) Set booting mode, (**b**) access point, (**c**) screen device configuration. Source: authors.

The infrastructure we developed is open source and available at the “Neurodevices” (accessed on 20 August 2024) repository on GitHub. It was designed to streamline the operation of various sensor devices through a set of unified MQTT services. When a device powered by this firmware initializes, its configuration status is checked. If not configured, after activating boot mode for 10 s (Figure 4a), it generates a Wi-Fi network (via the access point or AP, in Figure 4b) that, upon connection, opens a captive portal for configuration, as shown in Figure 4c.

Once configured, the device establishes three MQTT topics: one to receive service requests, another to send status responses, and a third to transmit data. Our specific adaptation of this library involved integrating services to start and stop transmitting data from the insole’s pressure sensors.

The updated firmware allows data retrieval via MQTT by sending a JSON request that specifies the experiment duration and sampling frequency to the command topic. Subsequently, the client subscribes to the transmission topic to receive data.

To prevent network overload, the device autonomously halts data transmission after the specified experiment duration, a critical feature for managing several devices. This MQTT-based approach ensures efficient and reliable communication suitable for IoT devices, capitalizing on its lightweight protocol, asynchronous messaging capabilities, and resilience to network fluctuations and bandwidth constraints.

### 2.6. Experimental Setup

**Figure 5:**
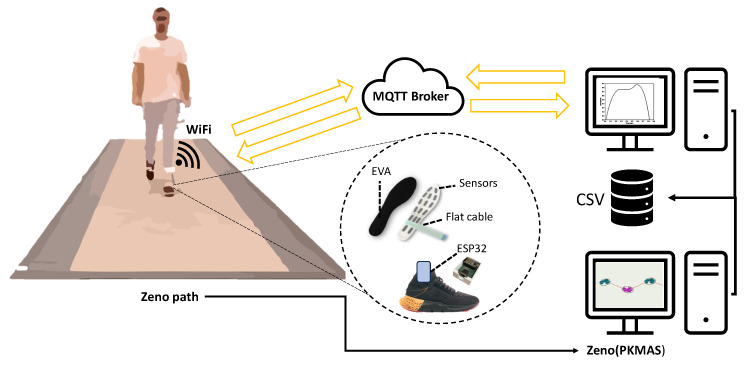
Experimental setup abstract. Source: authors.

The acquisition device was securely attached to the volunteer’s foot and shank to ensure comfort during use. The volunteers were instructed to walk at their usual pace along a 10-m Zeno^®^ sensor platform, as shown in Figure 5. Data were collected along the six mid-walk meters along the 10-m path. The initial and final two-meter walks were identified as the acceleration and deceleration zones, respectively.

Three trials were carried out for each individual. Vertical ground reaction forces (vGRF) data from five gait cycles were used to analyze the stance and swing phases of the gait cycle.

### 2.7. Data Processing

The pipeline for all data processing and graph plots was performed offline using Python with Numpy, Pandas, and Matplotlib libraries. Initially, a Savitzky–Golay noise filter was applied to reduce the noise in the signal. It is essential to highlight that the analysis was performed after offline data normalization using min–max scaling, as illustrated in Equation (1).

(1) $$x_{\mathrm{norm}}=\frac{x-\min(X)}{\max(X)-\min(X)}$$

(2) $$x_{\mathrm{Insole}}=\frac{insole-\min(insole)}{\max(insole)-\min(insole)}$$

(3) $$x_{\mathrm{Zeno}}=\frac{Zeno-\min(Zeno)}{\max(Zeno)-\min(Zeno)}$$

The data were extracted from all devices (Zeno and insole) and organized into CSV files. After applying the normalization method, the vertical ground reaction forces (vGFR) graphics were plotted using Python libraries. To synchronize the gait cycles between each device, the first contact identified by the images, the time, and the record of peak force was used.

### 2.8. Statistical Analysis

After data processing, statistical analyses were conducted using the Scipy libraries in the Visual Studio Code^®^ programming environment (version 1.91.1). The Pearson correlation coefficient was employed to determine the linear relationship between the developed system and the gold standard system. Additionally, Bland–Altman analysis was applied to assess the level of agreement between the two systems.

To clarify whether the developed system could capture the same range of data variation as the gold standard, the data were also presented with the mean and standard deviation. The analysis included all four participants, and the results are summarized in Section 3.

The Shapiro–Wilk test was conducted to assess the normality of the data. Since the data did not follow a normal distribution, the Wilcoxon test was chosen as an alternative non-parametric method to analyze the differences between the systems.

## 3. Results

**Figure 6:**
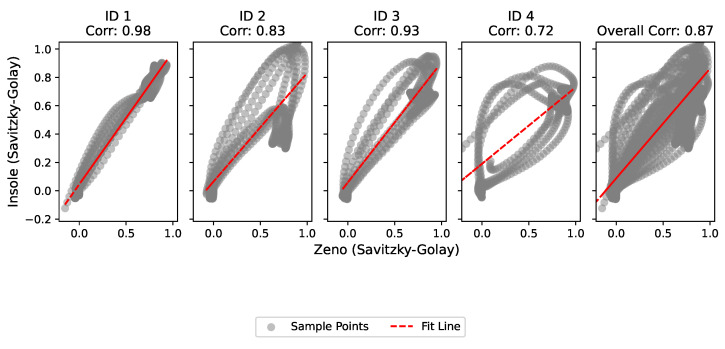
Pearson’s correlation plots between the insole and Zeno systems. Source: authors.

The descriptive statistics are shown in Table 2. The mean value of the Pearson correlation coefficient calculated between the insole and the Zeno^®^ (PKMAS) during four gait cycles for all volunteers resulted in r=0.87, as illustrated in Figure 6, with the highest value attributed to volunteer 1 (ID 1) r=0.98 and the lowest value to volunteer 4 (ID 4) r=0.72. Values of the calculated correlation coefficient (r) above 0.9 represent excellent statistical correlation, between 0.75 and 0.9 represent good statistical correlation, between 0.5 and 0.75 moderate statistical correlation, and below 0.5 poor statistical correlation [30,31].

**Figure 7:**
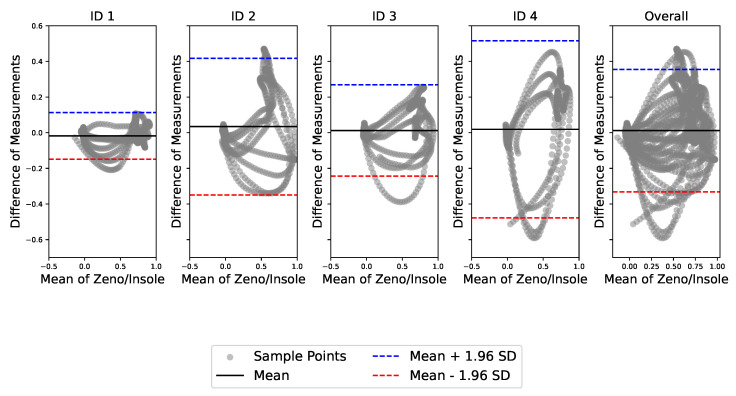
Bland–Altman plots. Source: authors.

The agreement between the systems was assessed using a Bland-Altman plot, as shown in Figure 7. The overall mean difference between the measurements of the two systems was approximately 0.011. The limits of agreement were determined to be −0.405 to 0.407, indicating that 95% of the differences between systems measurements fell within this range.

**Figure 8:**
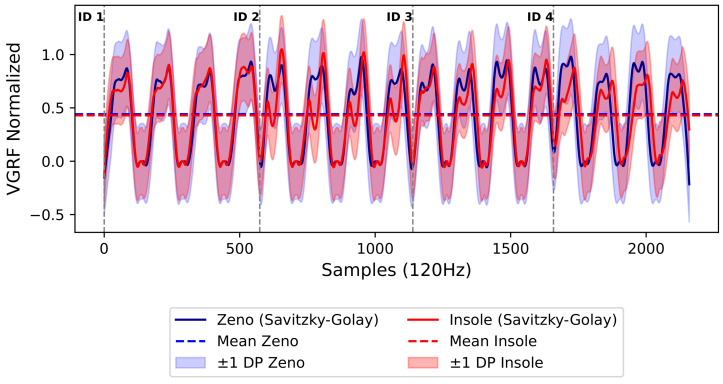
Mean and standard deviation of vertical ground reaction forces during four gait cycles with all volunteers (ID 1, ID 2, ID 3, ID 4), comparing the Insole and Zeno systems. Source: Authors.

In Figure 8, we present a detailed representation of the vGRF, mean, and standard deviation during walking for all volunteers. This illustration provides valuable information about sensor activation during walking, characterizing the dynamics of the load distribution.

These results lead us to believe that underlying factors may contribute to some discrepancies, and a more comprehensive exploration of the system limitations and potential areas for improvements are discussed in Section 4.

## 4. Discussion

The system was designed to extract and analyze gait kinetics, with the potential for structured interfaces with various wearable devices, including sensors and actuators. Our goal was to propose a system based on prefabricated insole pressure sensors and test it in an initial protocol, using the Zeno^®^ system (PKMAS) for performance comparison. This approach allowed us to evaluate new proof-of-concept applications for the IoT MQTT-based infrastructure and services we developed.

The Pearson correlation test revealed a significant relationship between the data obtained from both systems (r=0.87), indicating that the developed gait analysis system has the potential to characterize gait phases and subphases. The overall mean difference between the measurements of the two systems was approximately 0.011, suggesting that the bias between the measurements of the Zeno and Insole systems is small but significant. When analyzing the graphs for different subjects (ID 1 to ID 4), most of the points fell within the limits of agreement, indicating good agreement between the systems. However, greater dispersion was observed in the Bland–Altman plot mean values, indicating potential bias or variability in the measurements.

As shown in Figure 8, the vGRF curves of both systems were positively correlated. However, it is important to note that the Insole system exhibited reduced performance in preserving critical signal characteristics during the stance phase. Although both systems demonstrated high variability, the Insole system showed early or persistent activation at certain points, leading to unexpected peaks and valleys.

In the literature, there is still no consensus on the best sensor placement points [32]. We believe that factors such as using only six pressure points may have affected the quality of the signal reconstruction. Additionally, the force-resistive sensors can be significantly influenced by factors such as the flexure, the insole position within the shoe, the type of shoe, and the thickness of the socks [33,34].

New material proposals may be an alternative to address these issues. Other research groups are already investing in the development of advanced materials such as graphene, carbon fiber, triboelectric, and in vivo (fungi) [35,36,37,38,39], demonstrating the great evolutionary potential of these devices; however, they are still far from the reality of most research centers and public hospitals as implementable systems.

Concerns about the accuracy of insoles have already been described in the literature. Deberardinis et al. [40] worked on researching transfer functions concerning different insole sizes that improved the observed errors by up to 10%, making them more reliable for clinical and research applications. Other authors have explored machine learning and deep learning techniques for reconstructing signals [41,42,43,44].

A positive aspect of the system is its support for a data acquisition rate of 120 Hz, the same used by the Zeno^®^ system (PKMAS). We consider this sampling rate acceptable, given that the available systems range from 25 Hz to over 750 Hz [42]. The system was also able to transmit data effectively and maintain the device charge during the required autonomy period. However, challenges related to network stability, such as connection loss, were encountered during data collection.

Compared to other similar approaches in instrumented insoles [43,45,46], the developed system stands out for maintaining a low cost, a discreet and comfortable design, connectivity, and low power consumption using prefabricated sensors and an MQTT-based infrastructure. However, issues related to accuracy and robustness, especially regarding the signal reconstruction and sensor response under different wearing conditions, still need improvement.

The system developed in this study shows promise as a valuable tool, particularly for physiotherapists and rehabilitation specialists, in assessing gait disorders and monitoring treatment progress. The MQTT-based communication infrastructure allows real-time data collection from multiple sensors, enabling treatment protocols across multiple devices.

As future steps, we plan to improve the system by incorporating the 16 available sensors using a controller board with the necessary pins and investigating issues related to possible signal interference due to sensor flexion inside the shoe. We also expect similar performance in clinical populations by adding more sensors, which may offer better spatial resolution and greater robustness in analyzing different gait patterns. Furthermore, we propose to use the system to study motor control in individuals with Parkinson’s disease by integrating the insole system with functional electrical stimulation (FES) and electroencephalography (EEG).

## 5. Conclusions

This study provided significant results that are in line with the existing literature on similar systems. The analysis of the data obtained from the insole system demonstrated a small but significant difference between the systems, still presenting a good correlation and agreement; however, further studies with a larger sample are necessary.

In addition, the communication infrastructure developed is promising and offers new possibilities for multi-device treatments and assessments in the future. The developed gait analysis system has the potential to become a valuable and cost-effective tool for healthcare professionals in the treatment and monitoring of disorders related to gait instability.

The results highlight the viability of the system for practical application and its ability to contribute significantly to gait analysis and monitoring, after improvements are made regarding the signal accuracy.

## Figures and Tables

**Table 1:** FS-INS-16Z Legact insole operational characteristics.

Characteristic	Value
Pressure range	0.5–10 kg
Action mode	Compression
Activation force	500 g
Activation time	<10 ms
Operating temperature	−20 °C to +65 °C
Response time	<10 ms

**Table 2:** Descriptive statistics and Wilcoxon signed-ranks test.

	N	Mean	SD	SE	Coefficient of Variation
Zeno	2160	0.441	0.372	0.008	0.843
Insole	2160	0.430	0.339	0.007	0.789
Wilcoxon signed-rank					
Measure 1	Measure 2	W	z	df	*p*
Zeno	Insole	732,384.000	5.411	-	<0.001

## Data Availability

The datasets presented in this article are not readily available due to time limitations. Requests to access the datasets should be directed to email: christian.rathke@edu.isd.org.br or victor.pimentel@escolar.ifrn.edu.br.
